# A Chinese telemedicine-dialogue dataset annotated for named entities

**DOI:** 10.1186/s12911-023-02365-3

**Published:** 2023-11-16

**Authors:** Shanshan Wang, Yajing Yan, Rong Yan, Ting Li, Kaijie Ma, Yani Yan

**Affiliations:** 1 School of Economics and Management, Yan’an University, Yan’an, China; 2 College of Computer Science-College of Software, Inner Mongolia University, Hohhot, China; 3 Medical School, Yan’an University, Yan’an, China; 4 School of Information Science, Beijing Language and Culture University, Beijing, China; 5 Obstetrics and Gynaecology Department, Peking University People’s Hospital, Beijing, China

**Keywords:** Telemedicine, Long multiword named entities, Medical dialogue, Temporal named entities

## Abstract

**Background:**

A large collection of dialogues between patients and doctors must be annotated for medical named entities to build intelligence for telemedicine. However, since most patients involved in telemedicine deliver related named entities in informal and long multiword expressions, it is challenging to tag their telemedicine dialogue data. This study aims to address this issue.

**Methods:**

With the telemedicine dialogue dataset for obstetrics and gynecology taken from haodf.com, we developed guidelines and followed a two-round procedure to tag six types of named entities, including disease, symptom, time, pharmaceutical, operation, and examination. Additionally, we developed four deep-learning models based on this dataset to establish a benchmark for named-entity recognition (NER).

**Results:**

The distilled obstetrics and gynecology dataset contains 2,383 consultations between doctors and patients, of which 13,411 sentences were from doctors, and 17,929 were from patients. With 63,560 named entities in total, the average number of characters per named entity is 4.33. The experimental results suggest that LatticeLSTM performs best on our dataset in terms of accuracy, precision, recall, and F score.

**Conclusion:**

Compared with other datasets, this dataset offers three novel facets. This study offers intricately tagged long multiword expressions for medical named entities. Second, this study is one of the first attempts to mark temporal entities in a medical dataset. Third, this annotated dataset is balanced across the six types of labels, which we believe will play a considerable role in expanding telemedicine artificial intelligence.

## Background

Telemedicine refers to the practice of delivering patient care remotely via medical consultations. Over the years, telemedicine has become an increasingly vital complement to traditional face-to-face medicine. This can be attributed to several advantages, including facilitation of access to care for people living in areas short on medical services such as rural areas and reducing health care costs. Telemedicine has two integral parts thus far. One is the search engine, which enables people to obtain answers to their medical questions by entering some associated words or phrases in a search box. The other is the online platform facilitating conversations between patients and doctors and providing timely interventions accordingly. While telemedicine is inspiring, there are some limitations in its techniques. First, as most patients have no formal medical training, they may struggle to determine what medical expressions should be used in search engines to correctly retrieve answers. Second, telemedicine increases the risk of clinician burnout. To address such problems, telemedicine needs a large collection of well-annotated dialogues between patients and doctors as training data for named-entity identification and to build intelligence into its the two technical facets.

The medical dialogues occurring in telemedicine platforms, especially those in the fields of obstetrics and gynecology, often contain long multiple-word expressions for symptoms, diseases, medical history, etc. Multiple-word named entities can be composed of any words or characters, and their medical meaning cannot be traced back to their parts. For instance, the long multiple-word expression “  (After menstruation, I always drip brown discharge for about a week)” from a patient on haodf.com (https://www.haodf.com/) is a medical semantic unit, within which no one word adequately depicts its meaning. In natural-language processing, properly dealing with such a long multiple-word named entity is a novel and challenging task. Hence, to elaborate on this issue, we present a Chinese-telemedicine-dialogue dataset annotated for such complex multiple-word named entities with a focus on the obstetrics and gynecology specialties.

The obstetrics and gynecology dialogues in this study were taken from haodf.com (https://www.haodf.com/) and have been manually annotated according to BIO notations [1]. The tagged dataset is available at https://github.com/Yajing-bot/CTDD, and have six classes of named entities: disease, symptom, time, pharmaceutical, operation, and examination. To establish benchmarks for named-entity recognition (NER), we experimented with four deep-learning models on this distilled dataset.

In contrast with other Chinese medicine-dialogue datasets in telemedicine, this annotated dataset of obstetrics and gynecology offers three main advantages. First, most named entity units in patients’ statements display long multiword rather than phrase or word syntactic idiosyncrasies [2, 3]. Second, the temporal named entities have been labeled, which are currently missing in the telemedicine-dialogue literature. Third, the entities are balanced across the six categories. Because the collection of conversations between patients and doctors in electronic medical records are essentially different from those in telemedicine in terms of syntactic idiosyncrasies, similar work regarding the former is not included to compare with our study. In the latter, the medical conditions and history are delivered by patients in irregular and long expressions, whereas in the electronic medical record, as told by the patient, doctors report shorter expressions with medicine expertise.

## Method

### The notation scheme and tool for annotating

The BIO (Begin, Inside, Outside) notation scheme is adopted for annotating named entities in this study [1]. The purpose of the BIO scheme is to associate each word in a text with its corresponding named entity. It uses three labels to represent different parts of an entity: B (Begin), marking the starting position of an entity, the first word of the entity; I (Inside), representing the inside part of a named entity, the intermediate words of the entity; O (Outside), referring to the nonentity part, words that do not belong to any named entity. BIO is a commonly used NER tagging scheme due to its simplicity, intuitiveness, and ease of understanding. It provides a clear representation of the positions and boundaries for named entities in the text, providing the foundation for named-entity recognition tasks.

**Fig. 1:**
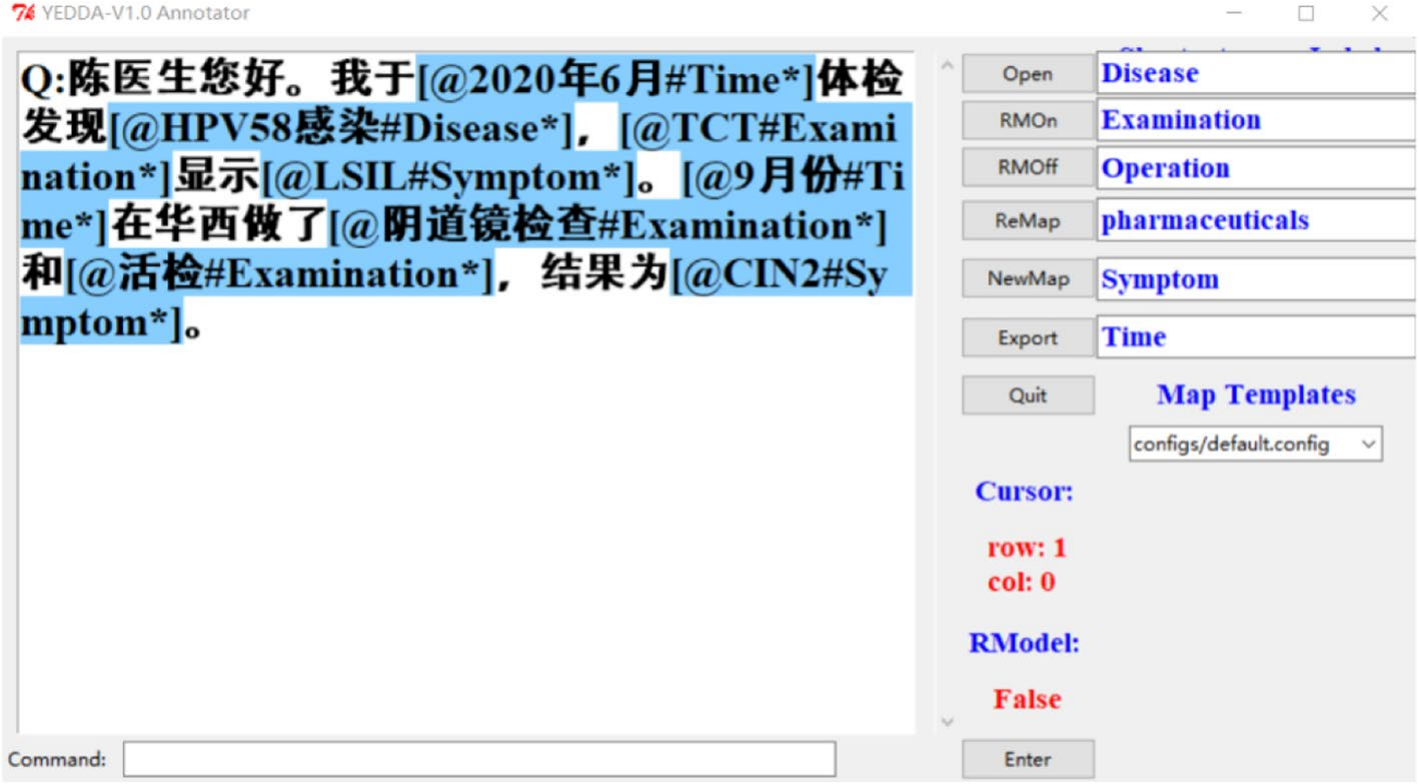
An exemplary tagging task supported by the YEDDA tool

During annotation, the YEDDA tool [4] was used to help annotators in tagging. An example tagging task supported by YEDDA is illustrated by Fig. 1. Each named entity is highlighted in blue, and the notation “#” is followed by the category each entity belong. Consider “  ” as an example. “Time” points out that “  ” is a temporal entity. Once the marking task for a dialogue is completed, the YEDDA tool automatically outputs a txt file, which contains the sequence of word-BIO label pairs for the dialogue.

### Development of the guidelines for annotating named entities

**Fig. 2:**
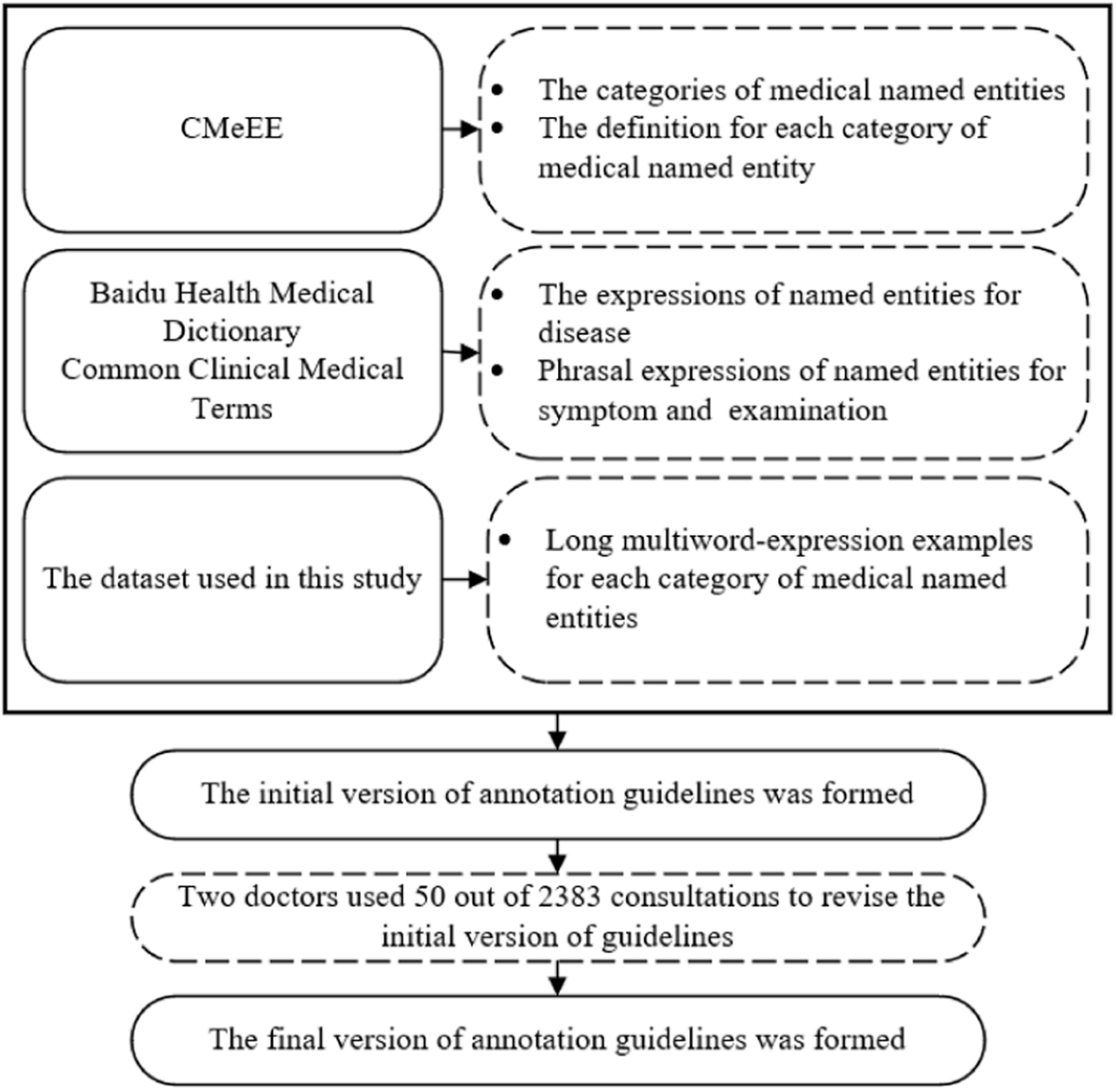
The development process of guidelines for annotating named entities

In addition to temporal named entities, other named entities can exactly locate their definitions in the UMLS (Unified Medical Language System) [5]. In the sentences from patients, expressions of time points such as time, date and time interval have been tagged as temporal named entities [3]. The entire marking process was performed under the guidelines we established to ensure quality and medical specificity. While setting the guidelines, the principles from Common Clinical Medical Terms (2019 Edition) (http://www.gov.cn/zhengce/zhengceku/2020-01/10/content_5467970.htm), CMeEE [6], and the Baidu Health Dictionary (https://jiankang.baidu.com/widescreen/home) were adopted. Moreover, two doctors in obstetrics and gynecology were involved in revising the guidelines with the aid of 50 of the 2,383 consultations [7]. The guidelines development is detailed in Fig. 2.

Following CMeEE, the classes of medical named entities in this study and their definitions were established in the initial version of the guidelines. Note that, in addition to long multiword named entities, there are also phrasal entities in the dataset. Thus, to more accurately label entities, the entity terms from Baidu Medical Dictionary and Common Clinical Medical Terms were matched to our dataset for annotating. Finally, based on the two doctors’ revisions, the final version of the annotating guidelines was formed.

### Construction of the dataset

 

**Fig. 3:**
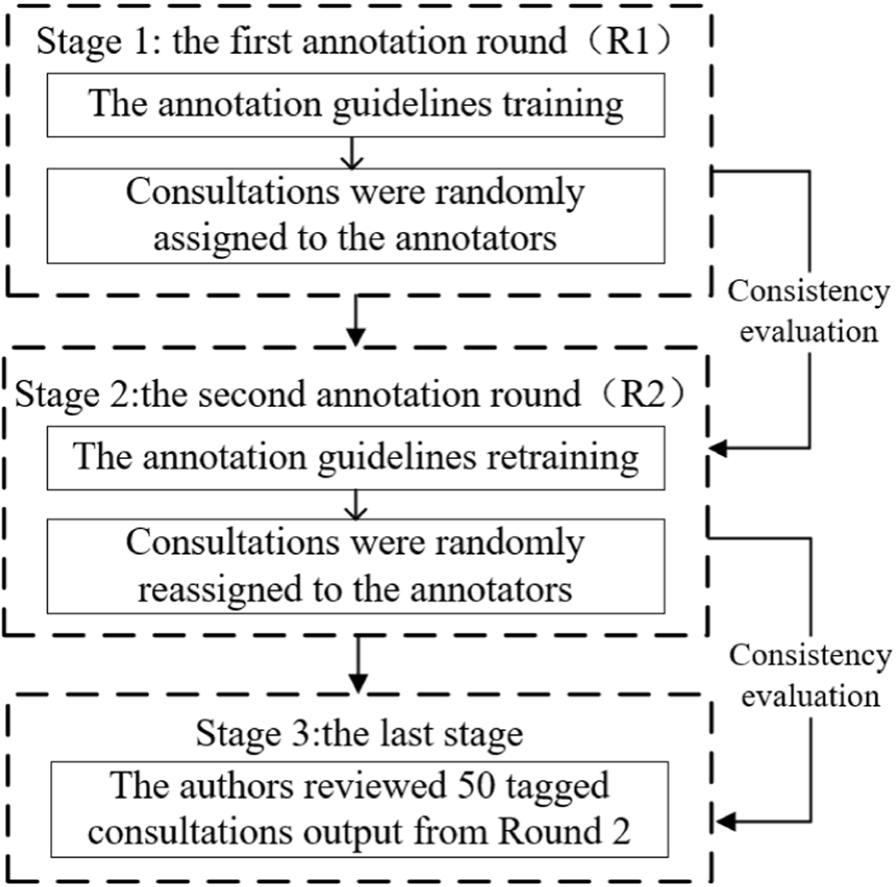
The annotation process

**Fig. 4:**
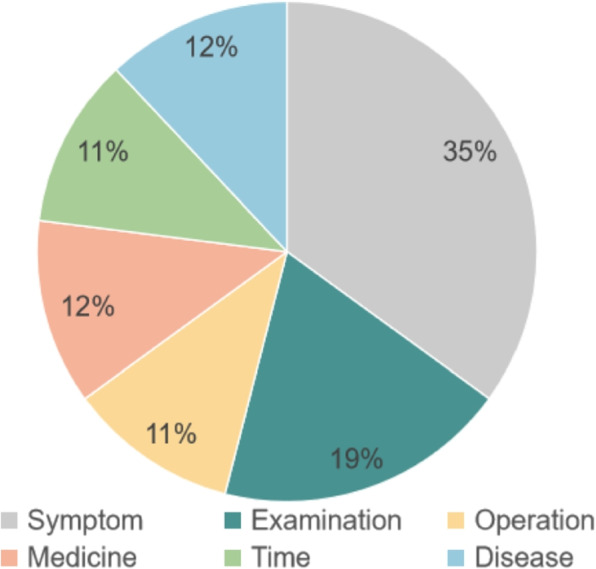
The proportion of each type of named entity to the overall occurrences of entities

- Data preparation: Our dataset consists of 2,383 Chinese consultations between doctors and patients, conducted from April 2013 to June 2022 on haodf.com (https://www.haodf.com/). The dataset is focused on the specialties of gynecology and obstetrics with a total of 31,340 sentences, 13,411 from doctors and 17,929 from patients. Each consultation covers descriptions of patients’ medical conditions as well as conversations between doctors and patients. The following fields are included in the data: details of the present disease, medical history, how long the symptom has persisted, the help needed from doctors, and diagnosis and treatment suggestions provided by doctors.
- Dataset annotation: This dataset was sorted into six classes of named entities: symptom, disease, examination, pharmaceutical, operation, and time. The entire annotation process followed a two-round procedure under the guidelines we established. While tagging, each batch of consultation was assigned to two annotators to handle in turn, each turn corresponding to a round (Fig. 3). The entities with ambiguous or intricate meanings were resolved according to the predominant vote among the paper’s authors. In the last stage, 50 of the tagged consultations from Round 2 were reannotated by us to examine the annotation quality carried out in Round 2.
- Dataset statistics: The following provides some statistical data on the annotated version of this dataset for comparison to other datasets. To the best of our knowledge, IMCS-NER, which is about the pediatrics specialty, is the only Chinese dataset on consultations in telemedicine. Hence, it is selected as the benchmark to evaluate the dataset presented in this paper. As shown in Table 1, the average count of characters contained in the named entities from our dataset is larger than that from IMCS-NER, as well as the average number of characters per consultation. In addition, the ratio of characters needed to be marked is higher in our dataset. Under these circumstances, it can be concluded that the treatment and future proper handling of the named entities from our dataset are fairly challenging. Except for those features, the two datasets reported in Table 1 are analogous in other measurements. For the proportion of each kind of named entity to the overall occurrences of entities, Fig. 4 reveals that our dataset is fairly balanced across labels. This can alleviate the issues brought by downstream tasks conducted on unbalanced data.

### Consistency evaluation

Consistency is used to evaluate the degree of agreement among observers in a uniform phenomena. If multiple annotators achieve the required degree of agreement in the same data, then we can assume that they are independently responsible for their own labeling task and that the dataset composed of their labeling results meets the reliability requirement. In the area of named-entity tagging, F value and its related metrics such as precision and recall [8, 9] are popular for evaluating consistency between annotators in the same data. The specific approach is to treat the annotation result of one annotator (such as Round 1 in this study) as the standard answer and calculate the precision and recall of the annotation result of another annotator, and then calculate the F value. The corresponding formulae are given in Equations 1 to 3.

**Table 1:** Comparison with other existing dataset

	This dataset	IMCS-NER
Count of all named entities	63,560	74,698
Average length of entity	4.33	2.63
Count of total characters	1,700,392	1,621,161
Ratio of tagged characters to total ones	16.2%	12.1%
Average count of characters per consultation	713.55	589.04

Two-round annotation is utilized to yield gold standard tagging. The agreement rates between the two rounds are given on the values of precision, recall and F-measure [10, 11] in Table 2. Each of them is defined below.

1 $$\begin{aligned} \text {Precision} = \frac{\text {Count of characters from the named entities with same labels in Rounds}\ 1\ \text {and}\ 2}{\text {Total count of named}-\text {entity characters in Round}\ 2} \end{aligned}$$

2 $$\begin{aligned} \text {Recall} = \frac{\text {Count of characters from the named entities with same labels in Rounds}\ 1\ \text {and}\ 2}{\text {Total count of named} - \text {entity characters in Round}\ 1} \end{aligned}$$

3 $$\begin{aligned} \text {F-measure} = \frac{2 \times \text {Precision} \times \text {Recall}}{\text {Precision} + \text {Recall}} \end{aligned}$$

In Table 2, compared to Round 1, precision, recall and F-measure in Round 2 improve slightly. However, all values approach 0.9, which can be considered good consistency rates, especially the F measure. Note that when computing the values of precision, recall and F measure for Round 2, in the last stage, 50 consultations retagged by the authors were used as the denominator in Equation 1, and named-entity characters in Round 2 was used as the denominator in Equation 2.

**Table 2:** Consistency rates for Rounds 1 and 2

	Precision	Recall	F-measure
Round1	0.856	0.870	0.863
Round2	0.892	0.901	0.896

## Experiments

### Models

For future comparison, the four most popular deep-learning models in Chinese named-entity recognition were benchmarked on our dataset. The four models adopted here are BiLSTM-CRF [12], LatticeLSTM [13], BERT-CRF [14] and LeBERT-CRF [15]. During the experiments, these models’ parameters remain the same as those set in the studies that developed them. Additionally, the word embeddings for those models were initialized using both ctb.50d.vec [16] and gigaword_chn.all.a2b.uni.ite50.vec [17].

**BiLSTM-CRF** This model is a variety of long short-term memory (LSTM)-based models for sequence tagging. The bidirectional LSTM component can efficiently use both past and future context, while the CRF layer can use long-sequence tag information. The BiLSTM-CRF model has 300 hidden units and performs better than the previous state-of-the-art model on POS, chunking, and NER datasets and has less dependence on word embedding [12].

**LatticeLSTM** This model was specifically developed for Chinese named-entity recognition. Compared with character-based and word-based NER methods, the model has the advantage of leveraging explicit word information over character sequence labeling without suffering from segmentation errors. Four types of vectors are integrated into the model, including input vectors, output hidden vectors, cell vectors and gate vectors. The hidden size of the model is set to 200 [13].

**Bert-CRF** This model consists of BERT and CRF components. BERT is a bidirectional transformer encoder with large-scale language pretraining [14].

**LeBert-CRF** This model is a lexicon-enhanced BERT for Chinese sequence labeling, which includes external lexicon knowledge in BERT layers by a lexicon-adapter layer. The model is constructed with 12 transformer layers and is initialized using the Chinese-BERT checkpoint from Hugging Face. The lexicon adapter between the first and second transformers in BERT fine tune both BERT and pretrained word embedding while training [15].

### Results

In this study, we have experimented on our dataset for medical named-entity recognition with techniques from BiLSTM-CRF, LatticeLSTM, BERT-CRF, and LeBERT-CRF. The dataset is divided into training, validation, and test sets according to the ratio 8:1:1. This section presents the accuracy, precision, recall and F scores obtained by training those models on BIO labels in our dataset. Table 3 summarizes the results. Among these models, LatticeLSTM performs best in all scores. This may be because LatticeLSTM was specifically developed for Chinese named-entity recognition.

**Table 3:** The experimental results on our dataset

Models	Accuracy	Precision	Recall	F
BiLSTM-CRF	-	0.53	0.52	0.53
LatticeLSTM	0.90	0.57	0.55	0.56
Bert-CRF	-	0.46	0.50	0.47
LeBert-CRF	0.57	0.53	0.53	0.54

## Discussion

In addition to the dataset presented in this paper, two other medical dialogue datasets in Chinese have been built, namely, MedDialog-CN [18] and IMCS-NER [2]. However, MedDialog-CN, which was also culled from the haodf.com platform, lacks annotations for named entities. The named entities in IMCS-NER are biased to simple and shorter expressions in contrast to our dataset. The possible explanation for this bias is that IMCS-NER and our dataset cover different medical specialties: IMCS-NER is about pediatrics, and our dataset is about obstetrics and gynecology. Due to the differences between medical areas, compared with pediatrics, the clinical descriptions of obstetrics and gynecology exhibit complex and longer expression characteristics. For example, “  (Have chocolate-color striped discharge during menstruation)” is a long multiword expression for menstruation symptoms. In comparison, the named entities in IMCS-NER are often presented as short phrases such as “  (fever)” and “  (cough violently)”.

Another difficulty exhibited by telemedicine datasets is that most patients are not well trained with medical knowledge. Therefore, they deliver named entities in irregular and varying ways. Even for named entities with the same semantic sense, different patients may deliver it using distinguishing utterances. For instance, the same symptom has been described by some patients with different lexical items like “  (After menstruation, I always drip brown discharge for about a week)”, and “  (The period of menstruation was prolonged each time, and after the fourth or fifth day of the menstruation cycle, coffee-color spotting appeared).”

Under the abovementioned conditions, we found that it is confusing to identify the start and end positions of named entities in our dataset. Under the guidelines proposed in this study, each exemplar expression illustrated in this paper has been treated as a single symptom entity. We also believe that even in datasets like IMCS-NER, by tagging long expressions for medical named entities, more exact medical information can be discovered. For instance, the entity of “  (Coughing intermittently)” is more meaningful than just “  (Cough).”

The dataset presented in this paper may arouse new medical intelligence issues for researchers, including long multiple-word named-entity recognition, and low-frequency symptom information extraction. Furthermore, since extracting temporal entities is essential for medical information retrieval [2], time intervals and points such as “  (7th of October)”, “  (Three months ago)”, and “  (For over half a month)” were marked as temporal named entities in this research. To date, temporal named entities are currently missing in the Chinese telemedicine dataset. On the whole, the dataset provided in this paper can promote intelligence growth for telemedicine, which further benefits doctors and geographically distant patients.

## Conclusion

In this study, we manually annotated a Chinese dataset for medical named entities based on telemedicine dialogues between doctors and patients. This dataset about the specialties of obstetrics and gynecology contains 2,383 dialogues and 31,340 sentences in total. The named entities in this dataset are categorized into six classes: disease, symptom, time, pharmaceutical, operation, and examination, and this is one of the first attempts to label long multiword named entities and temporal named entities from telemedicine dialogue. Moreover, our dataset is balanced on the named-entity labels, and experimental results have been achieved on such deep-learning models as BiLSTM-CRF, LatticeLSTM, BERT-CRF, and LeBERT-CRF to construct a benchmark for future comparison. This dataset may play a considerable role in expanding Chinese telemedicine artificial intelligence.

## Data Availability

The dataset is publicly available at https://github.com/Yajing-bot/CTDD.

## Abbreviations

BIO: B-begin, I-inside, O-outside
UMLS: Unified Medical Language System
POS: Position
NER: Named-entity recognition
BERT: Bidirectional encoder representation from transformers
LSTM: Long short-term memory
CRF: Conditional random field

## References

[1] Ramshaw LA, Marcus MP. Text Chunking Using Transformation-Based Learning. In: Armstrong S, Church K, Isabelle P, Manzi S, Tzoukermann E, Yarowsky D, editors. Natural Language Processing Using Very Large Corpora. Text, Speech and Language Technology, vol. 11. Dordrecht: Springer; 10.1007/978-94-017-2390-9_10.

[2] Chen W, Li Z, Fang H, Yao Q, Zhong C, Hao J, et al. A benchmark for automatic medical consultation system: frameworks, tasks and datasets. Bioinformatics. 2023;39(1):btac817. 10.1093/bioinformatics/btac817. 10.1093/bioinformatics/btac817 PMC9848052 36539203

[3] Zhou Y, Yan Y, Han R, Caufield JH, Chang K-W, Sun Y, et al. Clinical Temporal Relation Extraction with Probabilistic Soft Logic Regularization and Global Inference. In: Proceedings of the AAAI Conference on Artificial Intelligence, vol. 35, issue no. 16. 2021 p. 14647–14655. 10.1609/aaai.v35i16.17721.

[4] Yang J, Zhang Y, Li L, Li X. YEDDA: A Lightweight Collaborative Text Span Annotation Tool. In Proceedings of ACL 2018, System Demonstrations. Melbourne: Association for Computational Linguistics; 2018. p. 31–36.

[5] Bodenreider O (2004). The Unified Medical Language System (UMLS): integrating biomedical terminology. Nucleic Acids Res..

[6] Hongying Z, Wenxin L, Kunli Z, Yajuan Y, Baobao C, Zhifang S. Building a Pediatric Medical Corpus: Word Segmentation and Named Entity Annotation. CLSW. 2020. p. 652–664.

[7] Cai L, Wang ST, Liu J-H, Zhu Y-Y (2020). Survey of data annotation. J Softw..

[8] Carletta J. Assessing Agreement on Classification Tasks: The Kappa Statistic. Comput. Linguist. 1996;22(2):249–54.

[9] Hripcsak G, Rothschild AS (2005). Agreement, the f-measure, and reliability in information retrieval. J Am Med Inf Assoc..

[10] Yang J, Guan Y, He B, Qu C-Y, Yu Q-B, Liu Y-X, Zhao Y-J (2016). Corpus construction for named entities and entity relations on chinese electronic medical records. J Softw..

[11] Albright D, Lanfranchi A, Fredriksen A, Styler WF, Warner C, Hwang JD, Choi JD, Dligach D, Nielsen RD, Martin J (2013). Towards comprehensive syntactic and semantic annotations of the clinical narrative. J Am Med Inform Assoc Jamia..

[12] Huang Z, Xu W, Yu K. Bidirectional LSTM-CRF Models for Sequence Tagging. CoRR abs/1508.01991. 2015. p. 1–9.

[13] Zhang Y, Yang J. Chinese NER Using Lattice LSTM. ACL. 2018;(1):1554–64.

[14] Huang K, Altosaar J, Ranganath R. ClinicalBERT: Modeling Clinical Notes and Predicting Hospital Readmission. CoRR abs/1904.05342. 2019. p. 1–6.

[15] Liu W, Fu X, Zhang Y, Xiao W. Lexicon Enhanced Chinese Sequence Labeling Using BERT Adapter, issue no. 1. ACL/IJCNLP. 2021. p. 5847–5858.

[16] Xue N, Xia F, Chiou FD, Palmer M (2005). The penn chinese treebank: Phrase structure annotation of a large corpus. Nat Lang Eng..

[17] Mikolov T, Sutskever I, Chen K, Corrado GS, Dean J. Distributed Representations of Words and Phrases and their Compositionality. NIPS. 2013. p. 3111–3119.

[18] Zeng G, Yang W, Ju Z, Yang Y, Wang S, Zhang R, et al. MedDialog: Large-scale Medical Dialogue Datasets. In: Proceedings of the 2020 Conference on Empirical Methods in Natural Language Processing (EMNLP). Association for Computational Linguistics. 2020. p. 9241–9250.

